# Risk factors associated with progressive myelomalacia in dogs with complete sensorimotor loss following intervertebral disc extrusion: a retrospective case-control study

**DOI:** 10.1186/s12917-019-2186-0

**Published:** 2019-12-03

**Authors:** Aude Castel, Natasha J. Olby, Hongyu Ru, Christopher L. Mariani, Karen R. Muñana, Peter J. Early

**Affiliations:** 1 Department of Small Animal Clinical Sciences, University of Tennessee Veterinary Teaching Hospital, College of Veterinary Medicine, Knoxville, TN 37966-4544 USA; 2 Department of Clinical Sciences, College of Veterinary Medicine, North Carolina State University, 1060 William Moore Drive, Raleigh, NC 27607 USA; 3 Comparative Medicine Institute, North Carolina State University, 1060 William Moore Drive, Raleigh, NC 27607 USA

**Keywords:** Ascending-descending myelomalacia, Spinal cord injury, Intervertebral disk disease, Paraplegia

## Abstract

**Background:**

Progressive myelomalacia (PMM) is a usually fatal complication of acute intervertebral disc extrusion (IVDE) in dogs but its risk factors are poorly understood. The objective of this retrospective case-control study was to identify risk factors for PMM by comparing dogs with complete sensorimotor loss following IVDE that did and did not develop the disease after surgery. We also investigated whether any risk factors for PMM influenced return of ambulation. Medical records of client-owned dogs with paraplegia and loss of pain perception that underwent surgery for IVDE from 1998 to 2016, were reviewed. Dogs were categorized as PMM yes or no based on clinical progression or histopathology. Walking outcome at 6 months was established. Signalment, onset and duration of signs (categorized), steroids, non-steroidal anti-inflammatory drugs (yes or no), site of IVDE (lumbar intumescence or thoracolumbar) and longitudinal extent of IVDE were retrieved and their associations with PMM and walking outcome were examined using logistic regression.

**Results:**

One hundred and ninety seven dogs were included, 45 with and 152 without PMM. A 6-month-outcome was available in 178 dogs (all 45 PMM dogs and 133 control dogs); 86 recovered walking (all in the control group). Disc extrusions at the lumbar intumescence were associated with PMM (*p* = 0.01, OR: 3.02, CI: 1.3–7.2). Surgery performed more than 12 h after loss of ambulation was associated with PMM (OR = 3.4; CI = 1.1–10.5, *p* = 0.03 for 12-24 h and OR = 4.6; CI = 1.3–16.6, *p* = 0.02 for the > 24 h categories when compared with the ≤12 h category). Treatment with corticosteroids was negatively associated with PMM (OR: 3.1; CI: 1.3–7.6, *p* = 0.01). The only variable to affect walking outcome was longitudinal extent of IVDE (OR = 2.6; CI = 1.3–5.3, *p* = 0.006).

**Conclusion:**

Dogs with lumbar intumescence IVDE are at increased risk of PMM. Timing of surgery and corticosteroid use warrant further investigations. PMM and recovery of walking are influenced by different factors.

## Background

Progressive myelomalacia (PMM) is one of the most sinister complications associated with thoracolumbar intervertebral disc extrusion (TL IVDE) [1, 2]. In this condition, there is progressive ascending and/or descending hemorrhagic necrosis of the cord following acute, severe thoracolumbar spinal cord injury (SCI) due to acute IVDE [2]. There is no current treatment for this devastating and typically fatal condition [2]. A definitive diagnosis requires post mortem evaluation of the spinal cord, but a presumptive diagnosis with a high clinical suspicion can be made based upon neurologic examination findings corroborated with magnetic resonance imaging (MRI) characteristics [3–6]. The clinical signs consistent with PMM are a combination of complete sensorimotor loss in the pelvic limbs and the tail, loss of spinal reflexes in the pelvic limbs, loss of abdominal tone and advancement of the caudal border of the cutaneous trunci muscle reflex (CTMR) [5, 6]. As the disease spreads to the thoracic and cervical spinal cord, progression to tetraparesis, loss of reflexes in the thoracic limbs, bilateral Horner’s syndrome and respiratory distress often arise prior to death or humane euthanasia [5]. Most dogs develop signs consistent with PMM within 48 h of presentation but although the majority progress and are euthanized within 3 days after onset of signs, a delayed progression can be observed [5].

The prevalence of PMM is 2% when all dogs with TL IVDE are considered [6] but it is dramatically higher in paraplegic pain perception negative dogs, ranging from 9 to 17.5% [6–10]. Breed appears to be important because a prevalence of 33% was reported in French bulldogs [11]. Risk factors associated with the development PMM have been reported in a relatively small cohort of PMM dogs, and, when considered separately, include a more severe neurologic status, younger age, site of disc extrusion at L5-L6, speed of onset of less than 24 h and the ratio of the hyperintensity on T2 weighed magnetic resonance image to the length of L2 [6]. However, when considered together in a multivariate analysis, the T2 weighted hyperintensity ratio was the only significantly significant predictor of PMM development. There is a need to examine a larger population of dogs in order to confirm risk factors implicated in the previous univariate analysis [6], and to identify new risk factors that illuminate the underlying pathophysiological mechanisms and potential therapies. Given the prevalence of PMM is highest in paraplegic pain perception negative dogs, we identified this population for additional examination. We focused on dogs undergoing surgery because, in this particular population, post-operative follow up was available and clinical progression could be documented to assess whether or not the dog developed PMM. The goal of this exploratory, retrospective study was to identify risk factors for the development of PMM in dogs with complete loss of motor function and pain perception in the pelvic limbs and tail undergoing a hemilaminectomy following thoracolumbar IVDE. The second goal was to evaluate whether risk factors for PMM also influenced long-term recovery of ambulation (with and without recovery of pain perception).

## Results

One hundred and ninety seven cases met our inclusion criteria (Additional file 1). Of these, 45 were included in the PMM group and the rest of the dogs (152/197) were included in the control group. In the PMM group, 12 dogs had a diagnosis of PMM confirmed at necropsy while the other dogs (33/45) had a presumptive diagnosis of PMM based on a combination of clinical signs and imaging findings. All but two of the 45 dogs in the PMM group were euthanized. The two remaining dogs showed halting of PMM after initial progression. Both remained paraplegic without pain perception, and one of them had progressed to develop paresis of the thoracic limbs. The clinical characteristics of the PMM dogs were described in a previous study [5]***.*** The detailed signalment for both groups is reported in Table 1. The most common breed in both groups was the Dachshund (19/45 in PMM group and 87/152 in the control group). There was no difference between breed representation, sex and age between the two groups (Table 1).

**Table 1:** Signalment of dogs included in the PMM and control groups (univariate analysis)

	PMM group (n = 45)	Control group (*n* = 152)	P-value
Age (y) (median, range)	5 (2–14)	4.5 (2–14)	0.56
≤ 6 y	37	119	
> 6 y	8	33	
Sex			0.95
Female intact	4	13	
Female spayed	21	66	
Male intact	7	22	
Male neutered	13	51	
Breed			0.18
Dachshund	19	87	
Cocker Spaniel	3	8	
Other breeds	23	57	

*PMM* progressive myelomalacia; *y* years

The results of the multivariate analysis of risk factors for development of PMM are provided in Table 2 with additional detail on speed of onset of signs and duration of signs provided in Table 3. Breed, age, sex, site, time from onset of signs to loss of ambulation, time from loss of ambulation to surgery, treatment with NSAIDs and treatment with corticosteroids were all evaluated. Three of these variables affected the risk of development of PMM, location of the disc extrusion, timing of surgical decompression and use of corticosteroids (*p* = 0.01, *p* = 0.03 and *p* = 0.01 respectively). More specifically, dogs with IVDE affecting the intumescence had higher odds of developing PMM compared to the TL region (odd ratio (OR) = 3.0, confidence interval (CI) = 1.3–7.2). A longer time to surgery was also associated with higher odds of PMM (OR = 3.4; CI = 1.1–10.5 for 12-24 h and OR = 4.6; CI = 1.3–16.6 for the > 24 h categories when compared with the ≤12 h category). Finally, dogs that had not received steroids had higher odds of developing PMM compared to dogs that had received steroids (OR = 3.1, CI = 1.3–7.6).

**Table 2:** Multivariate logistic regression in PMM (*n* = 45) and control groups (n = 152)) examining risk factors for PMM

	PMM	Control	P-value	OR	95% CI
Breed					
• Dachshund	19	87	0.08	0.49	0.2–1.1
• Cocker Spaniel	3	8	0.84	0.85	0.2–3.9
• Other	23	57	Ref		
Sex					
• Female intact	4	13	0.38	1.9	0.5–9.6
• Female spayed	21	66	0.40	1.5	0.6–3.4
• Male intact	7	22	0.63	1.33	0.4–4.1
• Male neutered	13	51	Ref		
Age					
• ≤ 6 y	37	119	0.8	1.13	0.4–3.0
• > 6 y	8	33	Ref		
Site					
• Intumescence	15	21	**0.01**	3.0	1.3–7.2
• Thoracolumbar	30	131	Ref		
Extent of extrusion					
• Extensive (> 1 disc space)	20	62	0.8	1.1	0.5–2.3
• Focal	25	90	Ref		
Treatment with NSAIDs					
• No	29	106	0.76	0.89	0.4–2.0
• Yes	16	46	Ref		
Treatment with steroid					
• No	34	86	**0.01**	3.1	1.3–7.6
• Yes	11	66	**Ref**		
Time from onset of clinical signs to loss of ambulation			0.62	See Table 3 for details	
Time from loss of ambulation to surgery			**0.03**		

*PMM* progressive myelomalacia, *OR*: odds ratio, *CI* confidence interval, *Ref* reference, *NSAID* non-steroidal anti-inflammatory drug

**Table 3:** Results of the logistic regression reported in Table 2 providing statistical details of categories of both timelines evaluated

	PMM	Control	P-value	OR	CI
Time from onset of clinical signs to loss of ambulation			0.62		
• ≤6 h	18	52	Ref		
• 6-12 h	10	29	0.54	0.73	0.3–2.0
• 12-24 h	7	28	0.18	0.45	0.1–1.4
• 24-48 h	5	16	0.29	0.49	0.1–1.8
• > 48 h	5	27	0.30	0.52	0.2–1.8
Time from loss of ambulation to surgery			**0.03**		
• ≤12 h	5	38	Ref		
• 12-24 h	24	59	**.03**	3.4	1.1–10.5
• > 24 h	16	55	**.02**	4.6	1.3–16.6

*PMM* progressive myelomalacia, *OR* odds ratio, *CI* confidence interval. *Ref* reference

A post hoc analysis was performed to examine whether the type of corticosteroid used (methylprednisolone sodium succinate versus other (dexamethasone, prednisolone sodium succinate and prednisone)) was important. When looking specifically at dogs that received methylprednisolone sodium succinate, there was no statistical significance between groups (*p* = 0.81). By contrast, there was still a significant difference between groups for dogs receiving other corticosteroids (p_adj_ = 0.02).

In the control group of 152 dogs that did not develop PMM, 6-month follow up was available in 133 dogs giving a total of 178 (including 45 dogs with PMM) dogs with an outcome. Of these, 86/178 (48%) regained the ability to walk by 6 months. In a small subset of dogs, long term follow-up was established over the telephone and in this instance, the presence of pain perception could not be determined so this number includes dogs with and without pain perception. All 45 dogs that developed PMM were defined as having failed to recover walking and so a total of 92/178 dogs (52%) did not recover (45 in the PMM group and 47 in the control group). The different risk factors (breed, age, sex, site, time from onset of signs to loss of ambulation, time from loss of ambulation to surgery, treatment with NSAIDs and treatment with corticosteroids) evaluated using multivariate logistic regression were assessed for their influence on the walking outcome at 6 months (Table 4). The only factor shown to have an influence on long term outcome was the longitudinal extent of the disc herniation (*p* = 0.006) with dogs with an extensive disc herniation being less likely to walk at 6 months than dogs with a focal herniation (OR = 2.6; CI = 1.3–5.3).

**Table 4:** Multivariate logistic regression for 178 dogs with available long term outcome (PMM (n = 45) and control dogs (*n* = 133)) examining risk factors’ influence on recovery of the ability to walk at 6 months

	Walking	Not walking	P-value	OR	CI
Breed					
• Dachshund	49	45	0.85	1.07	0.52–2.19
• Cocker Spaniel	31	4	0.23	2.5	0.57–11.1
• Other	6	43	Ref		
Sex					
• Female intact	9	7	0.70	1.26	0.37–4.33
• Female spayed	34	45	0.20	0.61	0.29–1.29
• Male intact	13	13	0.62	0.77	0.28–2.12
• Male neutered	30	27	Ref		
Age					
• ≤ 6 y	66	75	0.8	1.1	0.48–2.54
• > 6 y	20	17	Ref		
Site					
• Intumescence	11	21	0.08	2.19	0.90–5.37
• Thoracolumbar	75	71	Ref		
Extent of extrusion					
• Extensive	26	48	**0.006**	2.6	1.3–5.3
• Focal	60	44	Ref		
Time from onset of clinical signs to loss of ambulation			0.48		
• ≤6 h	27	36	Ref	1.4	0.54–3.53
• 6-12 h	16	18	0.5	0.92	0.34–2.47
• 12-24 h	13	18	0.87	1.9	0.62–5.92
• 24-48 h	11	9	0.26	2.0	0.76–5.64
• > 48 h	19	11	0.16	6	
Time from loss of ambulation to surgery			0.8		
• ≤12 h	20	20	Ref	0.78	0.3–1.8
• 12-24 h	34	41	0.58	0.74	0.3–1.9
• > 24 h	32	31	0.53		
Treatment with NSAIDs					
• No	58	62	0.68	1.2	0.57–2.45
• Yes	28	30	Ref		
Treatment with steroid					
• No	48	63	0.1	1.8	0.8–3.8
• Yes	38	29	Ref		

*PMM* progressive myelomalacia, *OR* odds ratio, *CI* confidence interval, *Ref* reference, *NSAID* non-steroidal anti-inflammatory drug

## Discussion

In this exploratory, retrospective study we evaluated the factors associated with development of PMM and recovery of walking in 197 dogs with surgically treated TL IVDE. All dogs presented with the most severe grade of injury, paraplegia with loss of pain perception and 45 developed PMM. In this large population of severely injured dogs including 45 dogs with PMM, it was possible to explore risk factors that had been implicated previously, and to identify new factors worthy of investigation in prospective studies. We confirmed that disc extrusion at the level of the lumbar intumescence was a risk factor and also identified two potential therapeutic risk factors, delaying surgery more than 12 h after loss of ambulation and failure to treat with corticosteroids. Neither of these factors were significant when looking at long term recovery of walking, highlighting the possibility that different risk factors are at play when considering development of PMM and recovery of walking. Both of these risk factors deserve further evaluation.

We started by evaluating breed as a risk factor because there could be genetic influences on the severity of or response to spinal cord injury that might lead to improved understanding of, and therapies for PMM. Dachshunds and Cocker spaniels were split out based on their frequency in our study cohort, but neither were associated with significant risk compared with other breeds. The high frequency of both breeds in each group likely represented the high frequency of IVDE in the population served by our institution [7, 10]. French Bulldogs appear to be predisposed to PMM [11] but our study population included only 2 of these dogs, one in each group (PMM and control), making it impossible to evaluate.

A previous retrospective study of risk factors for PMM evaluated a large number of dogs with a range of presenting injury severity [6]. Using a univariate approach, the study identified severity of deficits, age (< 5.8 years), site (L5/6), speed of onset (< 24 h) and T2 hyperintensity ratios on MRI as risk factors [6]. However, once a multivariate analysis was performed including the key risk factors, only T2 length ratio remained significant [6]. This could have been due to the overwhelming effect of presenting injury severity on outcome and the relatively small number (13) of dogs with PMM. In our study, we chose to examine risk factors in pain perception negative dogs because of their predisposition for this condition and we found that age was not a risk factor in this population. This is likely explained by age being a risk factor for increased severity of clinical signs [12], with younger dogs more likely to have worse injury severity and thus being predisposed to PMM.

Although the most common sites of IVDE are close to the thoracolumbar junction [7, 12–14], we found that disc extrusion at the level of the intumescence (i.e. between L3 and L6) was associated with development of PMM. Indeed, the odds of developing PMM were 3 times higher in dogs with IVDE at the level of the intumescence. This finding is consistent with a previous study that identified IVDE at L5-L6 as a risk factor for PMM [6]. In humans, ischemic myelopathy can occur following a variety of abdominal and thoracic surgeries, but also following intervertebral disc herniation, due to interruption of the artery of Adamkiewicz (also called the great radicular artery). This artery is an important supplier of the ventral two thirds of the human spinal cord in the thoracolumbar region and its hairpin course may predispose it to occlusion [15]. An equivalent artery is present in approximately 50% of dogs arising on the left from the L5 spinal artery [16] and it has been hypothesized that damage to this artery that ultimately supplies the ventral spinal artery (VSA) may trigger ischemia in “watershed areas” between the VSA territory and that of the pial vessels causing the vascular injury cycle that generates PMM [2, 6, 17, 18]. It is also possible that larger proportion of grey matter to white matter at the intumescence creates more severe secondary injury cycle, again triggering development of PMM. By contrast, the longitudinal extent of disc material within the vertebral canal was not a risk factor for the development of PMM, refuting the hypothesis that PMM results only from extensive, Funkquist type III IVDE causing multilevel interruption of the spinal cord vasculature [2, 19]. It is notable however, that dogs with extensive disc herniations were significantly less likely to recover the ability to walk when compared with focal disc herniations.

Prior studies have reported that peracute onset of clinical signs of spinal cord injury represented a risk factor for the development of PMM [2, 6, 19]. However, when we examined the time from onset of signs to loss of ambulation, we found no difference in PMM incidence between groups. This finding suggests that a peracute loss of the ability to walk is not necessarily associated with the development of PMM, but might simply cause more severe spinal cord injury [20], thus predisposing to PMM. By contrast, dogs that received surgery less than 12 h after loss of ambulation had significantly lower odds of developing PMM than dogs that had surgery within 12–24 and 24–48 h respectively. This suggests that prompt surgical decompression might prevent the development of PMM. Our findings do not contradict a recent prospective study that did not find an association with timing of surgery and outcome (defined as recovery of walking) [8]. In our study, timing of surgery influenced the odds of development of PMM, but did not affect the outcome (walking yes or no) at 6 months. We hypothesize that rapid surgical decompression prevents or limits the pressure changes that have been postulated to be associated with PMM development by propelling blood and debris along the neuroaxis [2, 21] without affecting the primary and secondary injury mechanisms within the spinal cord enough to affect the ultimate long term recovery if PMM does not develop.

We were interested to examine whether NSAIDs or corticosteroids might influence the development of PMM based on results of a previous clinical trial [10] and on the oxidative stress and marked inflammation in malacic cord segments [22]. Given the retrospective nature of this study, specific details of drugs and dosing were not always available, so they were categorized broadly as NSAIDs or corticosteroids. In our study population, we found no effect of NSAID administration but, quite unexpectedly, we found that corticosteroid administration prior to presentation reduced the odds of PMM development. Given the observations from our previous clinical trial in which the group of dogs receiving MPSS had a low rate of PMM [10], we examined the effect of MPSS administration alone, and found no protective effect. When we looked at the effect of other corticosteroids (excluding dogs that had received only MPSS), the results were significant. Unfortunately, due to the lack of detail on the type and doses of corticosteroids, it is difficult to assess the importance of these results and they should be treated with caution. Nevertheless these results warrant further investigation especially given that nowadays, corticosteroids have been mostly abandoned from treatment recommendations for dogs with SCI and particularly IVDE due to the lack of proven efficacy for recovery of walking and known side effects [10, 23–27]. As for timing of surgery, we may have uncovered an important reason to consider corticosteroid use in severely injured dogs and further studies are needed to determine whether or not this class of drugs could be beneficial in a very specific population of dogs at higher risk of PMM. Indeed, the observation that prompt surgery and steroid use are associated with lower odds of developing PMM lead us to consider certain pathophysiological mechanisms including intrathecal and intraparenchymal pressure changes as well as the role of the inflammatory cascade and other events such as endothelin-1 overexpression [28, 29], a target that can be modulated by corticosteroids.

There are several limitations to our study due to its retrospective nature. A large number of cases did not meet our inclusion criteria (initial neurological examination performed and documented by a board-certified-neurologist or neurology resident, imaging and surgery and follow up at our institution performed with a documented neurological examination at least 2 weeks after the initial presentation) due to missing data in their medical record, which could have affected our study population. Some dogs were euthanized on presentation without further diagnostic evaluation due to the severity of their initial neurologic status. This could have resulted in preferential exclusion of those dogs in which there was a clinical suspicion of PMM based on their presenting signs in particular. Determination of the speed of onset of signs was based on the history provided by the owners or the primary care veterinarian. All studies of this type have to rely on owners for this information, but as previously reported, these data will include inaccuracies [30]. However, this applied to all dogs in the study, not one specific subgroup. The exact type of drug treatment received was unclear in some medical records. For example, records indicated that the dog had received “steroids” with no further details on the type and dose. Lacking this important information, it was therefore difficult to investigate the effect of any specific corticosteroid other than methylprednisolone on the development of PMM limiting our ability to make conclusions and weakening all observations on steroid use. We did not evaluate the influence of imaging modality used because there were no published data suggesting that imaging modality could influence development of PMM. Finally, our study was designed to examine risk factors for PMM development specifically, and walking outcome secondarily. As a result, data on final outcome was missing for some of these dogs, reducing the size of the study cohort for this particular goal.

## Conclusion

In this retrospective study on dogs with paralysis and loss of pain perception, we identified risk factors for the development of PMM. A disc herniation at the level of the lumbar intumescence was significantly associated with the development of this condition and use of corticosteroids and prompt surgical decompression might be protective. Further prospective studies are needed to confirm our findings but will be challenging to design in an effective and ethical fashion. Leveraging large amounts of patient data from multiple centers using a data collection system such as that initiated by CANSORT SCI might provide an alternative method of examining these important questions more rigorously [31].

## Methods

### Case selection

The medical records of all dogs that presented to our institution between 1998 and 2016 with paraplegia and loss of pain perception in both pelvic limbs and tail due to IVDE were reviewed. These medical records are not available to the public; owners consent to evaluation of medical records for research purposes. Records were identified from the canine spinal cord injury program database and by searching medical records using the key words “myelomalacia’, “ascending-descending myelomalacia”, progressive myelomalacia”, “paresis”, “paraplegia”, “hemilaminectomy” Absence of pain perception was defined as lack of conscious response (vocalization, looking at the origin of the stimulus, trying to bite or moving away from the stimulus) to heavy pressure applied to the pelvic limb digits or the tail with forceps. We excluded cases with only partial loss of pain perception i.e. when pain perception was only absent in one pelvic limb or the tail. To be included dogs had to have a complete medical record with a detailed initial neurologic examination by a board certified neurologist or a neurology resident, a diagnosis of acute IVDE made either by myelography, computed tomography (CT), or magnetic resonance imaging (MRI) and surgery performed.

Of these cases, dogs with histopathologically confirmed or presumptive PMM were identified based on criteria detailed in a previous study [5]. Briefly, a presumptive diagnosis of PMM was made based on a combination of specific clinical signs that demonstrated a mismatch between neuroanatomic diagnosis and site of IVDE. These signs included paraplegia with no pain perception in both pelvic limbs and tail, complete loss of spinal reflexes (patellar, withdrawal, perineal), loss of anal tone, loss of abdominal tone, cranial advancement of the caudal border of CTMR during hospitalization or a CTMR cut off more than two vertebral levels cranial to the site of disc extrusion [5]. Dogs’ clinical signs did not have to progress to tetraparesis and euthanasia or death to be diagnosed with PMM as long as clinical progression that fulfilled the above criteria occurred because there are unusual cases in which the PMM process halts [17]. However, if the only sign of progression was cranial advancement of the CTMR, this was not considered diagnostic of PMM due to the possibility of an alternative explanation such as acute focal vascular event or second IVDE [32]*.* Because a previous study [5] showed that progression of PMM could take up to 13 days following onset of signs, we excluded dogs without a proper follow up including a neurological examination (documented in the medical records) later than 2 weeks post-presentation or dogs euthanized (with no signs of progression) before that time.

### Data retrieval and categorization

Dogs were grouped as PMM yes or no. The following data were extracted from the medical record: breed, age, sex, site and extension of the disc extrusion based on imaging or necropsy findings, time from onset of signs to loss of ambulation, time from loss of ambulation to surgery, treatment with a non-steroidal anti-inflammatory drug or treatment with steroids prior to presentation. Type of steroid was noted if known. In cases that survived, the long term outcome i.e. whether or not the dog regained the ability to walk (with or witout pain perception) at 6 months, was also noted to assess whether any risk factor for PMM identified could also affect the long-term outcome. We elected to look at the breed as a risk factor as a previous study [11] reported that French bulldogs were 3 times more likely to develop PMM compared to Dachshunds. We elected to look at age and sex based on a study in which younger age was associated with increased risk of PMM [6]. We investigated the effect of the site of disc herniation because IVDE at L5-L6 has previously been reported as a risk factor for PMM [6] and because in our previous retrospective study in PMM dogs about a third of dogs had a disc herniation involving the lumbar intumescence [5]. We hypothesized that a disc extrusion affecting the lumbar intumescence was a risk factor for PMM compared with thoracolumbar discs. Longitudinal extent of disc extrusion was examined because previous study suggested that disc material distributed over multiple levels (Funquist type 3 disc herniations) was a predisposing factor for PMM [19]. Speed of onset of signs and time from paralysis to surgical decompression were investigated based on previous studies [6–8]. One of these studies reported that rapid onset of paralysis (< 24 h) was associated with an increased risk of PMM [6]. The other studies found that time to surgery did not influence the long term outcome (walking, yes or no) in deep pain negative dogs, although they did not examine risk factors for development of PMM [7, 8]. We hypothetized that speed of onset of signs was a risk factor for PMM. Because of the study by Henke et al., [21] in which increased spinal cord intraparenchymal pressure was postulated as a cause of PMM, we hypothesized that early surgical decompression, to reduce intraparenchymal and intrathecal pressure, would prevent the development of PMM without influencing the recovery of the ability to walk.

Finally, in a multicenter randomized controlled trial (RCT) of methylprednisolone sodium succinate (MPSS) and polyethylene glycol (PEG) in deep pain negative IVDE dogs, we noted a low rate of PMM in the MPSS group [10]. This RCT was not designed to have the statistical power to examine outcomes related to PMM but the data suggested that the question should be evaluated more closely. We were therefore interested to see if this finding was confirmed in a larger dog population looking at steroids in general as well as methylprednisolone more specifically and non-steroidal anti-inflammator drugs. We hypothesized that steroids, and more specifically methylprednisolone sodium succinate would not influence the development of PMM.

Data on these risk factors were categorized for analysis. Two breeds were identified, the Dachshund and the Cocker Spaniel, because of high representation in the PMM group and dogs were classified as Dachshund, Cocker spaniel or other. The distribution of data on age and site of disc extrusion were inspected in both groups. This resulted in classifying dogs as younger than or equal to 6 years of age, versus greater than 6 years of age, and disc location as thoracolumbar (TL) (any site between T9 and L2/3) versus lumbosacral intumescence (L3/4 to L6/7). A previous study suggested that extensive disc extrusion predisposed to PMM [2] and so discs were classified as focal (one disc space and associated vertebrae) or extensive (more than one disc space affected) based on the radiology report or necropsy. The time from onset of clinical signs (including pelvic limb weakness, ataxia, reluctance to jump, kyphosis or pain) to loss of ambulation (speed of onset) was determined based on the history. Due to the nature of the data (retrospective, owner description, animals not continuously monitored), this time was categorized as ≤6 h, 6–12 h, 12–24 h, 24–48 and > 48 h. The time from loss of ambulation to surgery (time to surgery) was established based on owners’ or veterinarian’s report of the timing of loss of the ability to walk and on the start time of surgery reported on the anesthesia form. This time was categorized as ≤12 h, 12–24 h and > 24 h. Treatment with steroids was categorized into any type of corticosteroid (yes or no), and then the analysis was repeated with it categorized as methylprednisolone sodium succinate (MPSS) or any other type of corticosteroid. In cases that survived, the outcome at 6 months was categorized as walking yes or no.

### Statistical analysis

Summary data were prepared on the signalment, site and extension of the disc extrusion, speed of onset, time to surgery and the treatment received prior to presentation. Statistical analysis was performed with JMP Professional 13 (SAS). A multivariate logistic regression was performed to model the relationship between the PMM status (Yes or No) and all other covariates. The probability modeled was PMM = Yes. A post hoc analysis of type of corticosteroid used was performed and Holm-Bonferroni sequential correction was performed to adjust for the effect of multiple comparison for these analyses. A *p*-value < 0.05 was considered statistically significant. The analysis was repeated for walking at 6 months (Yes or No).

## Supplementary information

**Additional file 1.**Table of data analyzed in this study. Details of all risk factors for each dog are provided.

## Data Availability

The datasets used and/or analyzed during the current study are available in the supplementary materials provided.

## Abbreviations

CI: Confidence intervals
CT: Computed tomography
CTMR: Cutaneous trunci muscle reflex
IVDE: Intervertebral disc extrusion
MPS: Methylprednisolone
MRI: Magnetic resonance imaging
NSAID: Non-steroidal anti-inflammatory drug
OR: Odds ratio
PMM: Progressive myelomalacia
SCI: Spinal cord injury
