# Supplementary material for: Risk factors associated with progressive myelomalacia in dogs with complete sensorimotor loss following intervertebral disc extrusion: a retrospective case-control study
Source: BMC Vet Res. 2019 Dec 3;15:433. doi: 10.1186/s12917-019-2186-0 (PMC6892155; doi:10.1186/s12917-019-2186-0)
Supplement: Supplementary file 1 — Additional file 1.Table of data analyzed in this study. Details of all risk factors for each dog are provided. [file 12917_2019_2186_MOESM1_ESM.pdf]

ID	PMM status	Breed	Age	Site	Onset of signs to loss of ambulation	Loss of ambulation to surgery	Steroids
1	No	Other	<6 yo	Thoracolumbar	2	4	Yes
2	No	Dachshund	<6 yo	Thoracolumbar	1	3	No
3	No	Dachshund	>6yo	Thoracolumbar	1	1	Yes
4	No	Dachshund	>6yo	Thoracolumbar	3	2	Yes
5	No	Dachshund	<6 yo	Thoracolumbar	1	1	No
6	No	Dachshund	<6 yo	Thoracolumbar	1	3	No
7	No	Other	<6 yo	Thoracolumbar	2	2	Yes
8	No	Dachshund	<6 yo	Thoracolumbar	2	3	No
9	No	Dachshund	<6 yo	Thoracolumbar	3	4	Yes
10	No	Dachshund	<6 yo	Thoracolumbar	5	2	No
11	No	Dachshund	<6 yo	Thoracolumbar	5	5	No
12	No	Dachshund	<6 yo	Thoracolumbar	1	3	No
13	No	Dachshund	<6 yo	Thoracolumbar	1	4	No
14	No	Dachshund	<6 yo	Thoracolumbar	1	3	Yes
15	No	Dachshund	<6 yo	Thoracolumbar	5	3	No
16	No	Other	<6 yo	Thoracolumbar	5	5	Yes
17	No	Dachshund	<6 yo	Thoracolumbar	1	2	No
18	No	Other	<6 yo	Thoracolumbar	2	2	No
19	No	Dachshund	<6 yo	Intumescence	1	2	No
20	No	Dachshund	>6yo	Thoracolumbar	5	5	Yes
21	No	Dachshund	<6 yo	Thoracolumbar	4	4	Yes
22	No	Dachshund	<6 yo	Thoracolumbar	4	2	No
23	No	Other	<6 yo	Thoracolumbar	3	4	Yes
24	No	Dachshund	<6 yo	Thoracolumbar	5	2	Yes
25	No	Other	<6 yo	Thoracolumbar	4	3	No
26	No	Cocker Spaniel	<6 yo	Thoracolumbar	1	2	No
27	No	Dachshund	<6 yo	Thoracolumbar	1	2	No
28	No	Dachshund	<6 yo	Thoracolumbar	4	3	No
29	No	Dachshund	<6 yo	Thoracolumbar	1	3	No
30	No	Dachshund	<6 yo	Thoracolumbar	5	5	No
31	No	Dachshund	<6 yo	Thoracolumbar	4	5	No
32	No	Other	>6yo	Thoracolumbar	2	4	Yes
33	No	Dachshund	<6 yo	Thoracolumbar	1	2	No
34	No	Dachshund	<6 yo	Intumescence	4	3	No
35	No	Dachshund	>6yo	Thoracolumbar	1	2	Yes
36	No	Dachshund	<6 yo	Thoracolumbar	2	4	Yes
37	No	Other	<6 yo	Thoracolumbar	1	2	No
38	No	Dachshund	<6 yo	Thoracolumbar	1	3	No
39	No	Dachshund	<6 yo	Thoracolumbar	1	4	Yes

40	No	Dachshund	<6 yo	Thoracolumbar	1	2	Yes
41	No	Dachshund	<6 yo	Thoracolumbar	2	3	Yes
42	No	Dachshund	<6 yo	Thoracolumbar	5	3	Yes
43	No	Dachshund	<6 yo	Thoracolumbar	1	2	No
44	No	Other	>6yo	Thoracolumbar	3	4	Yes
45	No	Dachshund	>6yo	Thoracolumbar	5	4	Yes
46	No	Dachshund	>6yo	Thoracolumbar	3	3	Yes
47	No	Dachshund	<6 yo	Thoracolumbar	1	3	No
48	No	Dachshund	<6 yo	Thoracolumbar	5	4	Yes
49	No	Dachshund	<6 yo	Intumescence	1	3	Yes
50	No	Other	<6 yo	Thoracolumbar	5	5	Yes
51	No	Dachshund	<6 yo	Thoracolumbar	1	3	Yes
52	No	Other	>6yo	Thoracolumbar	1	2	Yes
53	No	Other	<6 yo	Thoracolumbar	2	3	Yes
54	No	Dachshund	<6 yo	Thoracolumbar	1	5	No
55	No	Dachshund	<6 yo	Thoracolumbar	1	1	No
56	No	Dachshund	<6 yo	Thoracolumbar	2	5	Yes
57	No	Other	<6 yo	Thoracolumbar	2	3	Yes
58	No	Dachshund	<6 yo	Thoracolumbar	4	1	No
59	No	Dachshund	<6 yo	Thoracolumbar	3	3	No
60	No	Dachshund	>6yo	Thoracolumbar	1	3	No
61	No	Dachshund	>6yo	Thoracolumbar	3	4	Yes
62	No	Other	<6 yo	Thoracolumbar	3	4	Yes
63	No	Other	>6yo	Intumescence	3	3	No
64	No	Dachshund	>6yo	Thoracolumbar	3	3	Yes
65	No	Other	<6 yo	Thoracolumbar	3	4	No
66	No	Dachshund	<6 yo	Intumescence	1	3	No
67	No	Cocker Spaniel	<6 yo	Intumescence	2	3	Yes
68	No	Other	<6 yo	Thoracolumbar	2	3	No
69	No	Dachshund	<6 yo	Thoracolumbar	3	2	No
70	No	Dachshund	<6 yo	Thoracolumbar	1	1	No
71	No	Other	<6 yo	Intumescence	4	3	No
72	No	Dachshund	<6 yo	Thoracolumbar	3	4	No
73	No	Dachshund	<6 yo	Thoracolumbar	2	3	No
74	No	Dachshund	<6 yo	Thoracolumbar	5	4	Yes
75	No	Dachshund	>6yo	Thoracolumbar	1	4	Yes
76	No	Cocker Spaniel	<6 yo	Thoracolumbar	2	5	Yes
77	No	Dachshund	<6 yo	Thoracolumbar	2	3	Yes
78	No	Dachshund	<6 yo	Intumescence	4	4	Yes
79	No	Dachshund	>6yo	Thoracolumbar	1	3	No
80	No	Dachshund	<6 yo	Thoracolumbar	1	3	Yes
81	No	Other	<6 yo	Thoracolumbar	3	3	No
82	No	Other	<6 yo	Intumescence	2	3	Yes

83	No	Other	<6 yo	Thoracolumbar	3	5	Yes
84	No	Other	<6 yo	Thoracolumbar	3	4	Yes
85	No	Other	>6yo	Thoracolumbar	1	4	Yes
86	No	Dachshund	<6 yo	Thoracolumbar	4	4	No
87	No	Other	<6 yo	Thoracolumbar	3	4	No
88	No	Other	<6 yo	Thoracolumbar	1	3	No
89	No	Other	<6 yo	Thoracolumbar	1	3	No
90	No	Other	<6 yo	Thoracolumbar	3	4	No
91	No	Cocker Spaniel	<6 yo	Intumescence	4	2	No
92	No	Other	<6 yo	Thoracolumbar	2	3	No
93	No	Other	>6yo	Thoracolumbar	1	2	No
94	No	Other	<6 yo	Thoracolumbar	5	3	No
95	No	Other	<6 yo	Intumescence	3	4	Yes
96	No	Dachshund	>6yo	Thoracolumbar	1	3	No
97	No	Other	>6yo	Thoracolumbar	5	5	No
98	No	Dachshund	<6 yo	Thoracolumbar	2	4	Yes
99	No	Dachshund	<6 yo	Thoracolumbar	1	4	No
100	No	Other	>6yo	Thoracolumbar	5	3	No
101	No	Other	<6 yo	Thoracolumbar	4	4	Yes
102	No	Other	<6 yo	Thoracolumbar	2	3	Yes
103	No	Other	>6yo	Thoracolumbar	4	3	Yes
104	No	Other	>6yo	Thoracolumbar	1	3	Yes
105	No	Dachshund	>6yo	Thoracolumbar	5	4	No
106	No	Dachshund	<6 yo	Thoracolumbar	3	1	No
107	No	Dachshund	<6 yo	Thoracolumbar	4	3	Yes
108	No	Dachshund	<6 yo	Intumescence	3	4	Yes
109	No	Dachshund	<6 yo	Thoracolumbar	5	3	No
110	No	Other	<6 yo	Thoracolumbar	1	2	No
111	No	Dachshund	<6 yo	Thoracolumbar	1	3	No
112	No	Other	<6 yo	Intumescence	5	5	Yes
113	No	Dachshund	<6 yo	Intumescence	4	3	No
114	No	Cocker Spaniel	>6yo	Thoracolumbar	1	2	No
115	No	Dachshund	>6yo	Thoracolumbar	5	2	Yes
116	No	Other	>6yo	Thoracolumbar	3	5	No
117	No	Cocker Spaniel	<6 yo	Intumescence	2	3	Yes
118	No	Other	>6yo	Thoracolumbar	3	4	Yes
119	No	Dachshund	<6 yo	Thoracolumbar	5	2	No
120	No	Other	<6 yo	Thoracolumbar	2	3	No
121	No	Other	<6 yo	Thoracolumbar	3	5	Yes
122	No	Other	<6 yo	Thoracolumbar	1	3	No
123	No	Other	<6 yo	Thoracolumbar	2	2	No
124	No	Other	<6 yo	Intumescence	1	2	No
125	No	Other	>6yo	Thoracolumbar	3	2	No

126	No	Other	<6 yo	Thoracolumbar	2	5	Yes
127	No	Dachshund	<6 yo	Thoracolumbar	2	2	No
128	No	Dachshund	<6 yo	Thoracolumbar	5	4	No
129	No	Dachshund	<6 yo	Intumescence	2	4	No
130	No	Dachshund	<6 yo	Intumescence	2	3	No
131	No	Dachshund	<6 yo	Thoracolumbar	5	2	No
132	No	Other	<6 yo	Thoracolumbar	2	1	No
133	No	Other	<6 yo	Thoracolumbar	2	3	Yes
134	No	Cocker Spaniel	<6 yo	Thoracolumbar	1	3	No
135	No	Dachshund	<6 yo	Thoracolumbar	1	4	No
136	No	Other	<6 yo	Intumescence	1	3	Yes
137	No	Dachshund	<6 yo	Thoracolumbar	2	3	No
138	No	Dachshund	<6 yo	Thoracolumbar	3	4	Yes
139	No	Dachshund	>6yo	Thoracolumbar	5	4	Yes
140	No	Dachshund	<6 yo	Thoracolumbar	5	3	Yes
141	No	Other	>6yo	Intumescence	1	2	No
142	No	Other	>6yo	Intumescence	3	3	No
143	No	Other	<6 yo	Thoracolumbar	1	3	No
144	No	Other	<6 yo	Thoracolumbar	1	3	No
145	No	Cocker Spaniel	<6 yo	Thoracolumbar	4	3	No
146	No	Dachshund	<6 yo	Thoracolumbar	5	4	No
147	No	Other	<6 yo	Thoracolumbar	1	4	Yes
148	No	Dachshund	<6 yo	Thoracolumbar	3	2	No
149	No	Dachshund	<6 yo	Thoracolumbar	1	2	Yes
150	No	Other	>6yo	Thoracolumbar	5	5	Yes
151	No	Dachshund	>6yo	Thoracolumbar	1	4	Yes
152	No	Dachshund	<6 yo	Thoracolumbar	5	4	No
153	No	Other	<6 yo	Thoracolumbar	2	2	No
154	Yes	Other	<6 yo	Thoracolumbar	1	4	Yes
155	Yes	Dachshund	>6yo	Thoracolumbar	1	5	Yes
156	Yes	Dachshund	<6 yo	Thoracolumbar	5	4	No
157	Yes	Other	<6 yo	Thoracolumbar	3	3	Yes
158	Yes	Dachshund	<6 yo	Thoracolumbar	4	3	Yes
159	Yes	Dachshund	<6 yo	Intumescence	2	3	No
160	Yes	Other	>6yo	Thoracolumbar	2	3	No
161	Yes	Other	<6 yo	Thoracolumbar	2	4	No
162	Yes	Other	<6 yo	Thoracolumbar	4	3	No
163	Yes	Cocker Spaniel	>6yo	Thoracolumbar	1	3	No
164	Yes	Dachshund	>6yo	Intumescence	1	3	No
165	Yes	Cocker Spaniel	>6yo	Intumescence	2	3	No
166	Yes	Other	<6 yo	Intumescence	3	3	No
167	Yes	Other	<6 yo	Thoracolumbar	1	4	No
168	Yes	Other	<6 yo	Intumescence	4	3	No

169	Yes	Other	<6 yo	Intumescence	3	5	No
170	Yes	Dachshund	<6 yo	Thoracolumbar	1	2	No
171	Yes	Dachshund	<6 yo	Thoracolumbar	5	4	No
172	Yes	Dachshund	>6yo	Thoracolumbar	4	3	No
173	Yes	Other	<6 yo	Thoracolumbar	2	3	No
174	Yes	Other	<6 yo	Thoracolumbar	1	4	No
175	Yes	Other	<6 yo	Intumescence	5	5	No
176	Yes	Dachshund	<6 yo	Thoracolumbar	2	3	No
177	Yes	Other	<6 yo	Intumescence	2	3	Yes
178	Yes	Dachshund	<6 yo	Thoracolumbar	1	4	Yes
179	Yes	Dachshund	<6 yo	Thoracolumbar	5	5	Yes
180	Yes	Other	>6yo	Thoracolumbar	5	3	No
181	Yes	Dachshund	<6 yo	Intumescence	1	3	No
182	Yes	Dachshund	<6 yo	Thoracolumbar	1	4	No
183	Yes	Dachshund	<6 yo	Intumescence	3	4	No
184	Yes	Dachshund	<6 yo	Thoracolumbar	3	3	No
185	Yes	Dachshund	<6 yo	Intumescence	2	3	No
186	Yes	Other	<6 yo	Intumescence	2	4	No
187	Yes	Dachshund	<6 yo	Thoracolumbar	1	3	No
188	Yes	Other	<6 yo	Thoracolumbar	1	3	No
189	Yes	Other	<6 yo	Intumescence	4	3	No
190	Yes	Dachshund	<6 yo	Thoracolumbar	1	4	No
191	Yes	Dachshund	<6 yo	Thoracolumbar	1	3	No
192	Yes	Other	<6 yo	Intumescence	1	3	Yes
193	Yes	Cocker Spaniel	<6 yo	Thoracolumbar	1	2	No
194	Yes	Other	<6 yo	Thoracolumbar	1	3	No
195	Yes	Other	>6yo	Intumescence	1	3	Yes
196	Yes	Other	>6yo	Thoracolumbar	2	2	No
197	Yes	Other	<6 yo	Thoracolumbar	3	4	Yes

Key to time categories	
**≤6h**	1
**6-12h**	2
**12-24h**	3
**24-48h**	4
**> 48h**	5
